# Cystic Fibrosis Transmembrane Conductance Regulator Modulators in Cystic Fibrosis: A Review of Registry-Based Evidence

**DOI:** 10.3390/jcm14113978

**Published:** 2025-06-05

**Authors:** Donatello Salvatore, Angela Pepe

**Affiliations:** Cystic Fibrosis Center, AOR Ospedale San Carlo, 85100 Potenza, Italy; angela.pepe@ospedalesancarlo.it

**Keywords:** cystic fibrosis, CFTR modulator, registry, real-world evidence, ivacaftor, lumacaftor/ivacaftor, tezacaftor/ivacaftor, elexacaftor/tezacaftor/ivacaftor

## Abstract

Fibrosis transmembrane conductance regulator (CFTR) modulators (CFTRms) have significantly improved outcomes in people with cystic fibrosis (CF). Real-world evidence, particularly from national and international CF registries, is essential to assess their long-term effectiveness and safety. We reviewed published studies using registry data to evaluate the impact of CFTRms on clinical outcomes in individuals with CF. A narrative review of studies published between 2015 and 2025 was conducted, focusing on registry-based evaluations of ivacaftor, lumacaftor/ivacaftor, tezacaftor/ivacaftor, and elexacaftor/tezacaftor/ivacaftor. Primary outcomes included lung function, pulmonary exacerbations, nutritional status, and survival. Fifty-seven registry-based studies confirmed the benefits of CFTRms across diverse CF populations. Ivacaftor has demonstrated sustained improvements in forced expiratory volume in one second (FEV_1_), reduced exacerbations, and improved nutritional outcomes. Lumacaftor/ivacaftor and tezacaftor/ivacaftor have shown modest benefits, especially in homozygous F508del patients. The introduction of elexacaftor/tezacaftor/ivacaftor has led to unprecedented improvements in lung function and quality of life, along with a reduced need for lung transplantation. Methodological heterogeneity and incomplete data remain challenges. Registry data provide essential, complementary evidence to clinical trials and support the effectiveness of CFTRms in routine care. Continued efforts are needed to harmonize registry methodologies and outcome measures.

## 1. Introduction

Cystic fibrosis (CF) is an autosomal recessive genetic disorder caused by mutations in the cystic fibrosis transmembrane conductance regulator (CFTR) gene. These mutations result in dysfunction of the CFTR protein, leading to the production of thick, sticky mucus that affects multiple organ systems, particularly the lungs and digestive system. CF is characterized by chronic respiratory infections, progressive lung disease, pancreatic insufficiency, and other complications that significantly impact quality of life and reduce life expectancy [1,2].

The advent of CFTR modulators (CFTRms) has revolutionized CF treatment, shifting from symptom management to addressing the underlying molecular defect. These targeted therapies correct the function of the mutated CFTR protein and include potentiators (enhancing CFTR channel function) and correctors (improving CFTR protein processing and trafficking). Since the approval of ivacaftor (Kalydeco^®^) in 2012, subsequent developments have included combination therapies such as lumacaftor/ivacaftor (Orkambi^®^), tezacaftor/ivacaftor (Symdeko^®^/Symkevi^®^), and the triple-combination therapy elexacaftor/tezacaftor/ivacaftor (Trikafta^®^/Kaftrio^®^). These medications have demonstrated significant clinical benefits in randomized controlled trials (RCTs), including improvements in lung function, reductions in pulmonary exacerbations (PEx), enhanced nutritional status, and improved quality of life [1,2].

While controlled clinical trials provide valuable efficacy and safety data, they have limitations in generalizability due to their strictly defined conditions and selected patient populations. CF patient registries, established in many countries worldwide, offer complementary real-world evidence on the long-term effectiveness and safety of CFTRms across diverse patient populations. These registries systematically collect demographic, clinical, and treatment data from CF patients, enabling researchers to analyze outcomes in routine clinical practice and assess the impact of these novel therapies at a population level [3].

This review synthesizes published studies utilizing data from national and international CF registries to evaluate the real-world effectiveness, safety, and impact of CFTRms. By examining these registry-based studies, we provide a comprehensive overview of how these transformative therapies are affecting CF care and outcomes in clinical practice, identify potential disparities in access and response, and highlight gaps in current knowledge that warrant further investigation.

## 2. Materials and Methods

We conducted a narrative review of published studies using registry data to assess the impact of CFTRms. A literature search was performed using PubMed and EMBASE, covering the period from 1 January 2012 to 31 March 2025. The search strategy utilized a combination of Medical Subject Headings (MeSH) and keywords related to CF, CFTRms, and registries. This review included studies that utilized data from national and international CF registries to evaluate the real-world effectiveness, safety, and/or impact of CFTRms in individuals with CF. We included studies that reported on at least one of the following outcomes: pulmonary function, microbiology, clinical outcomes, epidemiology, gastrointestinal/nutritional outcomes, cystic fibrosis-related diabetes (CFRD), laboratory parameters, healthcare resource utilization, and fertility/pregnancy. We excluded studies that were not original research (e.g., reviews, editorials, and case reports), studies that did not utilize registry data (clinical trials and local cohorts), and studies that did not specifically evaluate CFTRms. Given the heterogeneity of study designs, interventions, and outcomes, we were unable to perform a meta-analysis. Instead, we synthesized the findings of the included studies narratively, grouping them according to major themes and specific CFTRm.

## 3. Results

This review identified 57 studies published between 2015 and 2025, with the majority (47/57, 82.5%) published from 2020 onwards, reflecting the growing body of real-world evidence following the introduction of these therapies. We categorized findings according to major themes and specific CFTRm (Table 1).

### 3.1. Pulmonary Outcomes

#### 3.1.1. Ivacaftor (IVA)

Hubert et al. examined 57 patients. Percent predicted FEV_1_ (ppFEV_1_) significantly improved from baseline at Year 1 (8.4%) and Year 2 (7.2%). Significant improvements were noted in BMI, reduced *P. aeruginosa* and *S. aureus* cultures, and decreased IV antibiotics and maintenance treatments. No significant adverse events occurred [4]. Merlo et al. reported durable clinical benefits during 5-year follow-up, with an annual ppFEV_1_ decline of −1.23 percentage points in the IVA cohort versus −2.03 in comparators, representing a 39% reduction in lung function decline with IVA treatment [5]. Szczesniak et al. found that IVA initiation reduced ppFEV_1_ variability (median reduction: 1.85), suggesting that this parameter may serve as a marker for therapeutic effectiveness [6].

#### 3.1.2. Lumacaftor/Ivacaftor (LUM/IVA)

Burgel et al. analyzed 827 patients stratified by baseline ppFEV_1_ into the following three subgroups: severe (<40), moderate (40–90), and normal (≥90). Treatment discontinuation was the highest in the severe group (28.9%). Significant ppFEV_1_ improvements were observed in the moderate (+2.9%) and severe (+0.5%) subgroups, but not in the normal subgroup. Intravenous antibiotic use decreased across all subgroups. Body Mass Index increased comparably across all subgroups [7]. Campredon et al. found that 1-year treatment with LUM/IVA was associated with a significant visual improvement in bronchial disease on chest Computed Tomography (CT) [8]. Their study also suggested that radiomics features on pre-therapeutic CT scans may help to predict lung function response under LUM/IVA, offering a potential pathway for personalized treatment approaches. Reix et al. observed discordant results between Lung Clearance Index (LCI), a marker of ventilation inhomogeneity, and ppFEV_1_ in one-third of patients, noting that LCI monitoring in adolescents and young adults with moderate lung disease gave more heterogeneous results than those reported in children with milder disease [9]. Zwitserloot et al. found that after 12 months of LUM/IVA, no significant change in ppFEV_1_ was observed in pediatric patients. However, significant improvements were demonstrated in small airway function (10.0% reduction in LCI) and structural lung disease markers (improved bronchus–artery analysis on CT scan). Additional improvements were noted in BMI, pulmonary exacerbation (PEx) frequency, and sweat chloride, with an efficacy comparable to phase 3 studies [10].

#### 3.1.3. Elexacaftor/Tezacaftor/Ivacaftor (ETI)

Martin et al. observed that in a large French cohort of lung transplant candidates with an F508del mutation, ETI treatment was associated with improvements in lung function (ppFEV_1_ + 13.4%), reductions in PEx (−86%), improved gas exchange, and enhanced nutritional status [11]. Most notably, lung transplantation could be deferred in most patients over a prolonged period, suggesting that access to triple-combination therapy could significantly decrease the number of lung transplantations in the CF population. Leo-Hansen et al. documented that ETI halts the progression of lung disease and improves ppFEV_1_ across all ages (+13%) and disease severities, with the annual rate of change improving from −1.4 ppFEV_1_ in the pre-treatment year to 2.7 ppFEV_1_ per year during treatment [12]. Cromwell et al. (2024) reported that extension of the ETI label to rare CFTR variants was associated with meaningful improvements in mean ppFEV_1_ (+3.9%) and a 45% reduction in PEx [13]. Drasbæk Philipsen et al. found a statistically significant, though not clinically relevant, increase in oxygen uptake after one year of ETI treatment among Danish patients [14].

### 3.2. Microbiology and Infection

#### 3.2.1. IVA

Frost et al. reported that long-term IVA use was associated with early and sustained reductions in *P. aeruginosa* rates (adjusted prevalence ratio, 0.68) via a combination of increased clearance in those with infection and reduced acquisition in those without infection. IVA use was also associated with a reduced prevalence of *Staphylococcus aureus* and *Aspergillus* spp., with implications for antibiotic stewardship and the potential to reduce the need for ongoing chronic antimicrobial therapy in this cohort [15]. Chesshyre et al. demonstrated a protective association between IVA use and Allergic Bronchopulmonary Aspergillosis (ABPA), representing the first study to identify this relationship. Their findings also showed that lower lung function, *Aspergillus* colonization, and Pa infection in children with CF were associated with the development of ABPA, highlighting the need for enhanced surveillance in these patients [16].

#### 3.2.2. ETI

In a cohort of 1092 people with CF, significant reductions in *Staphylococcus aureus* and *Pseudomonas aeruginosa* detection were observed up to 21 months after initiating ETI therapy. These reductions varied by pathogen, specimen type, age, baseline lung function, and pre-treatment colonization status, indicating that distinct biological mechanisms underlie the modulation of airway microbiota in response to ETI [17].

Pollak et al. found that one year after starting ETI, many people with CF who were initially positive for various CF-related pathogens shifted to a negative status; indeed, pre-ETI, 38.4% were positive for Pseudomonas aeruginosa (PsA) and 36.4% were positive for methicillin-sensitive *Staphylococcus aureus* (MSSA). Post-ETI, 38.7% of PsA-positive and 47.2% of MSSA-positive patients converted to a negative status. Similar improvements were observed for Burkholderia cepacia complex and Stenotrophomonas maltophilia [18]. These findings suggest that ETI reduces airway infections, with benefits extending into the second year of treatment, though some patients continue to carry these pathogens despite treatment.

#### 3.2.3. Multiple Modulators

Muhlebach et al. observed that factors predicting inhaled antibiotic prescription differed between 2011 and 2019, indicating changes in health and care for people with CF even prior to the introduction of triple modulators [19]. This suggests a shifting landscape in infection management concurrent with the development of CFTRm therapies.

### 3.3. Gastrointestinal and Nutritional Outcomes

#### 3.3.1. IVA

Calthorpe et al. reported that the licensing of IVA was followed by a lower prevalence of pancreatic enzyme replacement therapy (PERT) use in the eligible US population compared to the pre-licensing period, as well as a lower risk of PERT use in those who received treatment. Interestingly, they noted inconsistencies between US and UK CF registry data in this regard [20].

#### 3.3.2. ETI

Caley et al. investigated the mechanism behind BMI increases with ETI therapy, tentatively suggesting that this weight gain may not simply be attributable to an increase in oral intake [21]. Indeed, the pre- and post-ETI therapy group’s BMI significantly increased from 23.0 kg/m^2^ at baseline to 24.6 kg/m^2^ at follow-up, although there was a significant decrease in energy intake from 2551 kcal/day to 2153 kcal/day. They emphasized the need for further exploration into the underlying etiology of weight gain associated with ETI therapy.

#### 3.3.3. Multiple Modulators

Szentpetery et al. identified significant trends in Body Mass Index (BMI) across CF programs, reporting a relative decrease in underweight status by approximately 40% between 2000 and 2019, simultaneously with a >300% increase in overweight status and a >400% increase in obesity [22]. Patient-specific factors associated with a higher prevalence of obesity included an age of ≥46 years, living in a zip code with a median income of <USD 20,000, having at least one allele with a class IV or V mutation, ppFEV_1_ > 90, being prescribed IVA, and not being prescribed pancreatic enzymes. Patil et al. found that early height, but not early weight-for-length/BMI trajectories, may be associated with pubertal growth outcomes [23]. Their analysis indicated that CFTRm therapy shows the potential to improve pubertal growth outcomes, though they noted that further research is necessary.

### 3.4. Cystic Fibrosis-Related Diabetes (CFRD)

#### ETI

Nielsen et al. observed that in a Danish CF cohort, hemoglobin A1c (HbA1c) declined over 12 months of ETI treatment [24]. However, among a subset of patients with CFRD, they observed no changes in insulin usage and continuous glucose monitoring (CGM) glucose levels, suggesting differential effects on glycemic control depending on baseline diabetic status.

### 3.5. Laboratory Parameters

#### IVA and LUM/IVA

Gifford et al. reported that both IVA and LUM/IVA use were associated with higher hemoglobin levels in patients with CF, providing evidence of systemic effects beyond the pulmonary and gastrointestinal systems [25].

### 3.6. Healthcare Resource Utilization

#### 3.6.1. IVA

Kirwan et al. found that healthcare resource utilization decreased after commencing IVA treatment, based on an analysis of CF registry data from Ireland [26]. In the year after ivacaftor initiation, intravenous antibiotic treatment reduced by 46% and oral antibiotic treatment reduced by 49% This suggests potential economic benefits alongside clinical improvements. Granger et al. demonstrated a clear divergence in treatment patterns since the introduction of IVA in a number of key treatments widely used in CF [27]. They noted that further research is needed to investigate whether these differences in treatment patterns are associated with changes in health outcomes.

#### 3.6.2. ETI

Keogh et al. estimated that ETI is expected to result in a significant reduction in the total population requirement for intravenous antibiotics of between 16.1% (~17,800 days) and 43.6% (~39,500 days), contributing to an increased understanding of the changing healthcare needs of people with CF [28]. Råket et al. reported that in a national Danish cohort of people with CF, ETI was associated with substantial reductions in elective hospitalizations, elective outpatient contacts, the duration of elective hospitalizations, pharmacy visits, and blood sampling appointments, sustained for 2 years post-ETI initiation [29]. These findings highlight the real-world effectiveness of ETI in the context of a universal healthcare system. In another study, Råket et al. observed that two years after ETI initiation, reductions in the use of several routine therapies were observed in a national cohort of people with CF, with the largest declines in airway medications and antibiotics, highlighting ETI’s real-world impact beyond conventional clinical metrics [30].

### 3.7. Fertility and Pregnancy

#### Multiple Modulators

Heltshe et al. provided evidence of significantly increased numbers of pregnancies among women taking approved CFTRms, an important finding due to the unknown risks to pregnancy and fetal outcomes [31]. They suggested that these increases may have been temporary, possibly following pregnancy prevention during controlled clinical trials, or they may have resulted from altered perceptions about maternal survival with newly approved treatments.

### 3.8. Quality of Life

#### ETI

Martin et al. found that in a cohort of 331 individuals with advanced CF pulmonary disease initiating ETI, 65 were lung transplant candidates. After a median follow-up of 363 days, only 4 required listing or transplantation, while 61 no longer met the transplant criteria; ppFEV_1_ improved by 13.4% at one month and remained stable, with marked reductions in treatment burden, including 86% fewer intravenous antibiotics, 59% less oxygen therapy, and 62% less non-invasive ventilation. This highlights the comprehensive impact of modulator therapy beyond physiological parameters [32].

### 3.9. Adverse Events

#### ETI

Ramsey et al. reviewed data from clinical trials, post-marketing reports, an ongoing registry-based ETI post authorization safety study, and the peer-reviewed literature. They concluded that depression symptoms and depression-related events reported in people with CF treated with ETI were generally consistent with the background epidemiology of these events in the CF population and did not suggest a causal relationship with ETI treatment [33].

### 3.10. Epidemiology and Access

#### 3.10.1. IVA

McGarry et al. demonstrated that 5-year survival projections modeled for people with CF initiating IVA vs. standard care aligned closely with real-world registry data [34]. Their findings support the validity of modeling CF to predict long-term survival and estimate the clinical and economic outcomes of CFTRms.

#### 3.10.2. LUM/IVA

Sawicki et al. noted that LUM/IVA uptake was less rapid than that previously observed for IVA [35]. Age, insurance status, disease severity, and the use of other therapies differed in those prescribed LUM/IVA in the initial post-approval period. Rubin et al. projected that LUM/IVA in combination with standard care would increase median survival by 6.1 years compared to standard care alone, with greater gains observed when initiated at younger ages. Lifetime treatment beginning at ages 6, 12, 18, and 25 was associated with incremental median survival increases of 23.4, 18.2, 11.0, and 4.8 years, respectively, underscoring the importance of early and sustained therapy [36]. Kondratyeva et al. studied the efficacy and tolerability of LUM/IVA in patients with CF in Russia against the age aspect [37]. They found that in the 2-to-6-year-old age group, the achievement of normal sweat test values and the lowest number of adverse reactions were noted, while in the 12-to-18-year-old age group, positive dynamics were noted for a greater number of indicators, including an increase in FVC. Lindblad et al. reported that improvements in clinical status observed in people with CF treated with LUM/IVA were reflected in a reduced caregiver and societal burden in Sweden, noting that CF is associated with a high clinical, economic, and societal burden in that country [38].

#### 3.10.3. ETI

Stanojevic et al. projected that delayed access to ETI would have a negative impact on lung health and survival in the CF population [39]. Martin et al. found that ensuring access to triple-combination therapy could significantly decrease the number of lung transplantations in the CF population and has the potential to improve life expectancy [11]. Lopez et al. showed that treatment with ETI in pwCF homozygous for F508del resulted in a median projected survival of 71.6 years, representing gains of 23.2–33.5 years compared to prior therapies or best supportive care. Early initiation (ages 12–17) further increased projected survival to 82.5 years, suggesting that ETI may significantly extend life expectancy and reduce disease burden in this population [40]. Vega-Hernandez et al. observed a notable decline in the number of hospitalizations after CFTRm initiation in patients with CF in Wales, which appeared more pronounced in patients whose first CFTRm was ETI [41]. Hergenroeder et al. reported that while over 90% of eligible individuals were prescribed ETI within three years, time of first prescription was associated with demographic factors and disease severity. Earlier ETI initiation was associated with a greater disease severity and prior modulator use, while delayed access was linked to public insurance, high baseline lung function, Black race, and Hispanic ethnicity. They recommended further research to investigate the reasons for this delay and approaches to reduce time to initiation for ETI and future therapies [42].

#### 3.10.4. Multiple Modulators

Cobanoglu et al. found that approximately one quarter of the registered CF patients in Turkey were eligible for modulator drugs [43]. They noted that as frequent mutations in Turkish CF patients differ from those in North American and European CF patients, developing modulator drugs effective for these mutations is necessary. Furthermore, they emphasized that as modulator drugs are very expensive, financial support from the government in low- and middle-income countries like Turkey is noteworthy. Sanders et al. described the CFTRm-ineligible population in the US from 2017 to 2022 [44]. They emphasized that with a growing pipeline of therapies aimed at improving CFTR function in those who cannot benefit from modulators due to ineligibility, the characterization of both the size and outcomes of these populations is critical to inform optimal clinical development plans and future clinical trials. Guo et al. reported that an estimated 162,428 individuals are living with CF across 94 countries, of whom 65% are diagnosed and only 12% receive ETI. The burden is highest in countries lacking access to disease-modifying treatments, with substantial underdiagnosis—particularly in low- and middle-income regions—highlighting the need for expanded diagnostic and therapeutic coverage [45]. Their analysis showed the potential to improve rates of diagnosis and treatment for CF, so a higher percentage of patients receive the most effective triple-combination treatment. Tomlinson et al. found that both F508del prevalence and gross domestic product were associated with variable CFTRm usage rates, although a predominant reason was unclear as a result of poor consistency in registry reporting [46]. They called for urgent action to encourage the uniform reporting of registry data and increase the availability of novel CFTRm therapies to the global CF population. Kerem et al. highlighted that in a pan-European cohort of 47,621 individuals with CF, significant improvements in FEV_1_ % prediction and survival were observed over the past decade, particularly among children, adults, and those with the F508del mutation following the introduction of ETI. These gains were largely confined to higher- and middle-income countries, highlighting persistent disparities and the urgent need for improved access to care and therapies in lower-income regions.

Although CF treatment advancements have improved outcomes in many people with CF, these benefits are not evenly distributed globally [47]. They concluded that efforts to improve CF care in low-income countries, such as increasing awareness, ensuring access to therapies, and establishing specialized clinics, are essential to bridging this gap. Kondratyeva et al. focused on the genetic and molecular epidemiology of CF in the Russian population, utilizing data from the national CF registry, which provides valuable insights into regional variations in mutation patterns and potential modulator eligibility [48]. Erdal et al. similarly reported that only half of the patients registered in the Turkish CF registry were eligible for CFTRms, representing a significant difference from the CFTR variant profile seen in the USA and Europe [49].

### 3.11. Multiple Clinical Outcomes

#### 3.11.1. IVA

Sawicki et al., Bessonova et al., Kirwan et al., Volkova et al., Higgins et al., and Kawala et al. all reported favorable results across multiple clinical outcomes among IVA-treated patients [26,50,51,52,53,54]. These studies consistently support the concept that IVA functions as a disease-modifying therapy for CF, with Volkova et al. representing the largest longitudinal analysis of patients treated with ivacaftor in a real-world setting [52] In a 5-year longitudinal analysis, IVA-treated patients (*n* = 635) demonstrated significantly slower lung function decline (−0.7 vs. −8.3 ppFEV_1_), greater BMI gain (+2.4 vs. +1.6 kg/m^2^), and fewer exacerbations and hospitalizations compared to 1874 matched comparators. These real-world findings, consistent across US and UK cohorts, support the disease-modifying effects of CFTR modulation with IVA.

Salvatore et al. found that Italian patients with gating mutations are few and are characterized by milder phenotypes than F508del homozygous patients, with improved outcomes likely influenced by treatment with IVA [55].

#### 3.11.2. LUM/IVA

Kim et al. reported early favorable trends in clinical outcomes, including growth parameters, PEx, and hospitalizations, in children with CF who initiated LUM/IVA treatment between the ages of 2 and 5 years in the European CF Society Patient Registry (ECFSPR). In matched pediatric cohorts, treatment with lumacaftor/ivacaftor (LUM/IVA) led to significant improvements in BMI percentiles compared to F/MF and F/F comparators, with mean differences of 8.4 and 11.8, respectively. Additional gains in height and weight percentiles, along with reductions in pulmonary exacerbations and hospitalizations, further support the clinical benefits of LUM/IVA in children with CF [56].

Burgel et al. found that in a cohort of 845 patients initiating LUM/IVA, 18.2% discontinued treatment, primarily due to adverse events, with discontinuation being more likely in adults, those with ppFEV_1_ < 40%, and those with higher prior IV antibiotic use. Among those who maintained treatment, significant improvements in lung function, BMI, and reduced IV antibiotic use were observed, whereas those who discontinued treatment experienced clinical decline, underscoring the importance of treatment tolerability for sustained benefit [57].

#### 3.11.3. ETI

Burgel et al. found that in 245 patients with advanced CF (median ppFEV_1_ = 29), ETI led to a mean ppFEV_1_ increase of +15.1 and a mean weight gain of +4.2 kg. Marked reductions in supplemental care needs and lung transplant listings were observed, with a twofold decline in CF lung transplants by 2020, supporting the therapy’s capacity to significantly alter disease trajectory and defer transplantation [58]. Bower et al. reported that ETI treatment was associated with sustained clinical benefits, including a 79% reduction in pulmonary exacerbations, 74% reduction in hospitalizations, and mean ppFEV_1_ increases of +8.2 and +8.9 percentage points in Years 1 and 2, respectively. Improvements in BMI, reductions in pathogen prevalence, and substantially lower annualized rates of death (−72%) and lung transplantation (−85%) underscore the broad and durable impacts of ELX/TEZ/IVA in real-world settings [59].

#### 3.11.4. Multiple Modulators

Kondratyeva et al. analyzed the health status indicators of children with CF aged 6–18 years for 6 months of targeted therapy (LUM/IVA and ETI) in Russia [60]. This study provides a comprehensive analysis of the genetic and molecular epidemiology of CF in the Russian population, identifying 233 CFTR variants—47 of which are absent from international databases—and emphasizing their distinct genetic landscape compared to other European cohorts. The findings underscore the growing relevance of precise genetic diagnosis for personalized therapy, particularly with expanding CFTR modulator access, and highlight the need to evaluate the pathogenicity and therapeutic implications of novel and complex alleles.

**Table 1:** Studies included in the review.

TOPIC	Year	Registry	CFTR Modulator	Outcomes	Reference Number
Adverse events	2024	GER, US	ETI	Mental health	[33]
CF-related diabetes	2024	DK	ETI	HbA1c, CGM	[24]
Epidemiology and access	2018	US	LUM/IVA	Prescription of LUM/IVA	[35]
	2019	US	LUM/IVA	Simulation model, survival, survival projection	[36]
	2020	TUR	All	Allelic frequencies	[43]
	2021	CAN	ETI	Forecasting, microsimulation	[39]
	2022	Various	ETI	Diagnosed subjects, projections	[45]
	2023	UK	ETI	Survival	[40]
	2023	US	ETI	Survival, mortality	[34]
	2023	WAL	ETI	Hospitalizations	[41]
	2024	RU	All	Genetic variants	[48]
	2024	EU	All	FEV_1_, Gross National Income	[47]
	2024	Various	All	Prevalence, Gross domestic product	[46]
	2024	RU	LUM/IVA	Sweat chloride, FVC, ADR	[37]
	2024	SWE	LUM/IVA	Visits, costs, FEV_1_	[38]
	2025	US	ETI	Prescriptions, FEV_1_, ethnicity	[42]
	2025	US	All	Age, ethnicity, FEV_1_, prescriptions	[44]
	2025	TUR	All	CFTR variants	[49]
Gastrointestinal	2022	US	All	BMI	[22]
	2023	UK	ETI	BMI, energy intake	[21]
	2024	US, UK	IVA	PERT	[20]
	2024	US	All	BMI, Growth	[23]
Healthcare resource utilization	2019	IRL	IVA	Antibiotic cycles, hospitalizations, FEV_1_	[26]
	2022	UK	IVA	Burden of illness	[27]
	2022	UK	ETI	Burden of illness	[28]
	2025	DK	ETI	Hospitalizations	[29]
	2025	DK	ETI	Burden of illness	[30]
Imaging	2022	FRA	LUM/IVA	CT	[8]
Laboratory parameters	2019	US	IVA, LUM/IVA	Hgb levels	[25]
Microbiology	2024	GER	ETI	Culture positivity	[17]
	2019	UK	IVA	Culture positivity	[15]
	2025	UK	IVA	ABPA predictors	[16]
	2025	EU	ETI	Culture positivity	[18]
	2025	US	IVA, LUM/IVA	Culture positivity, treatments	[19]
Multiple clinical outcomes	2015	US	IVA	BMI, growth, FEV_1_	[50]
	2018	US	IVA	Hospitalizations, complications, FEV_1_, microbiology	[51]
	2020	US, UK	IVA	Hospitalizations, complications, FEV_1_, microbiology	[52]
	2020	US, UK	IVA	Hospitalizations, risks of death, transplant, PEx	[53]
	2020	FRA	LUM/IVA	FEV_1_, PEx, BMI	[57]
	2021	CAN	IVA	FEV_1_, PEx, BMI	[54]
	2021	ITA	IVA	FEV_1_, PEx, BMI	[55]
	2021	FRA	ETI	FEV_1_, PEx, BMI, treatments	[58]
	2023	US	ETI	PEx, hospitalizations, microbiology, BMI, FEV_1_	[59]
	2023	RU	ETI, LUM/IVA	FEV_1_, BMI	[60]
	2024	EU	LUM/IVA	Growth, PEx, Hospitalizations	[56]
Pregnancy	2017	US	IVA, LUM/IVA	Pregnancy rates and outcomes	[31]
Pulmonology	2018	FRA	IVA	FEV_1_, microbiology, treatments	[4]
	2021	FRA	LUM/IVA	FEV1, BMI, treatments	[7]
	2022	FRA	ETI	Rate of transplants, burden of illness	[11]
	2022	FRA	LUM/IVA	LCI, FEV_1_	[9]
	2024	DK	ETI	FEV_1_, FVC	[12]
	2024	DK	ETI	FEV_1_, BMI, oxygen uptake	[14]
	2024	US	IVA	FEV_1_	[5]
	2024	US	ETI	FEV_1_, PEx	[13]
	2025	NL	LUM/IVA	FEV1, LCI, CT scan, sweat chloride	[10]
	2025	US	IVA	FEV_1_ variability	[6]
Quality of life	2021	FRA	ETI	Treatment burden	[32]

CAN: Canada; DK: Denmark; FRA: France; GER: Germany; IRL: Ireland; ITA: Italy; NL: the Netherlands; RU: Russia; SWE: Sweden; TUR: Turkey; UK: United Kingdom; US: United States; WAL: Wales; CFTR: cystic fibrosis transmembrane conductance regulator; CGM: continuous glucose monitoring; FEV_1:orced_ expiratory volume in the 1st second; FVC: forced vital capacity; BMI: Body Mass Index; PEx: pulmonary exacerbation; LCI: Lung Clearance Index; CT: Computed Tomography; PERT: pancreatic enzyme replacement therapy, ABPA: allergic broncho-pulmonary aspergillosis.

## 4. Discussion

This comprehensive review of real-world evidence from national and international CF registries demonstrates that CFTRms have had a transformative impact on multiple aspects of CF care and outcomes. While the clinical benefits observed in RCTs have been largely confirmed in routine clinical practice, registry data provide valuable insights into the long-term effectiveness, safety profiles, and broader impacts of these therapies across diverse patient populations and healthcare systems.

The most consistent finding across registry-based studies is a significant improvement in lung function following the initiation of CFTRms, with a particular emphasis on the durability of these effects. For ivacaftor, studies by Hubert et al. [4] and Merlo et al. [5] confirm sustained benefits for up to five years post-initiation, with a notable slowing in the rate of lung function decline. The reduction in ppFEV_1_ variability identified by Szczesniak et al. represents a novel marker for therapeutic effectiveness that warrants further investigation [6].

For LUM/IVA, Burgel et al., Kim et al., and Zwitserloot et al. demonstrated efficacy across various baseline lung function levels and age groups, while highlighting the value of alternative assessment methods such as the Lung Clearance Index and BA analysis, particularly in pediatric populations, where FEV_1_ may have limitations as a marker of early disease [7,10,56]. The discordant results between the LCI and ppFEV_1_ observed by Reix et al. in one-third of patients further emphasize the importance of multiple assessment modalities, especially in adolescents and young adults with moderate lung disease [9].

The most dramatic pulmonary improvements were observed with triple-combination therapy (ETI), as evidenced by Burgel et al., Bower et al., and Leo-Hansen et al., who documented the halting of lung disease progression across all ages, all disease severities, and with prior modulator use [12,58,59]. The impact on lung transplantation candidates, as reported by Martin et al., represents a paradigm shift in end-stage disease management, with the potential to significantly reduce the need for transplantation in this population [11]. The extension of ETI to patients with rare CFTR variants, described by Cromwell et al., further expands the reach of these benefits to previously underserved populations [13].

Registry data have revealed significant reductions in respiratory pathogen burden following CFTRm therapy, most notably for Pa. The findings of Heltshe et al. and Frost et al. for IVA [15,16], alongside the more recent observations by Pollak et al. for ETI [18], suggest that effective CFTR modulation may reduce bacterial colonization through improved mucociliary clearance and an altered airway microenvironment. The protective association between IVA and ABPA identified by Chesshyre et al. adds a novel dimension to the antimicrobial benefits of these therapies, potentially extending to fungal complications of CF [17]. The evolving landscape of infection management in CF was further illustrated by Muhlebach et al., who observed changes in factors predicting inhaled antibiotic prescription between 2011 and 2019, even prior to the widespread use of triple modulators [19]. These microbiological improvements have important implications for antibiotic stewardship and may contribute to a reduced treatment burden and improved quality of life.

Registry studies have documented significant nutritional improvements with CFTRms, alongside potential reductions in pancreatic enzyme replacement therapy requirements, as noted by Calthorpe et al. [20]. However, the substantial increases in BMI observed in several studies raise important questions about the mechanisms involved and potential long-term implications. Caley et al. highlighted that weight gain with ETI may not be solely attributable to increased oral intake, suggesting possible metabolic effects that warrant further investigation [21].

The dramatic shift in CF nutritional epidemiology documented by Szentpetery et al., with marked decreases in underweight prevalence accompanied by substantial increases in overweight and obesity, represents a fundamental change in the nutritional management paradigm for CF [22]. The identification of patient-specific factors associated with a higher prevalence of obesity (including age of ≥ 46 years, lower socioeconomic status, class IV or V mutations, preserved lung function, ivacaftor prescription, and absence of pancreatic enzyme prescription) provides valuable guidance for targeted nutritional monitoring and intervention. The potential of CFTRms to influence growth trajectories, particularly during puberty, as suggested by Patil et al., represents an important area for future research, given the traditional challenges of growth impairment in CF [23]. Their finding that early height, but not early weight-for-length/BMI trajectories, may be associated with pubertal growth outcomes adds nuance to our understanding of growth dynamics in CF and highlights the potential of CFTRm therapy to positively impact this aspect of development.

The findings of Nielsen et al. regarding improvements in HbA1c with ETI therapy, albeit without corresponding changes in insulin requirements or glucose profiles in patients with established CFRD, highlight the complex relationship between CFTR function and glucose metabolism [24]. These observations suggest potential benefits for diabetes prevention rather than the reversal of established disease, which aligns with the current understanding of the progressive nature of pancreatic damage in CF. The association between CFTRms and increased hemoglobin levels reported by Gifford et al. reflects the systemic nature of the benefits derived from these therapies, extending beyond the respiratory and digestive systems typically associated with CF [25]. This finding underscores the importance of comprehensive assessments of modulator effects across multiple organ systems. Beyond the clinical endpoints and registry-based outcomes discussed in this review, a growing body of research has investigated laboratory biomarkers as tools for assessing the efficacy of CFTR modulator therapies. These biomarkers, particularly those measurable in blood, may serve as non-invasive, objective indicators of treatment response and disease activity. Several studies have explored the utility of inflammatory and epithelial injury markers, such as C-reactive protein (CRP), interleukin-1 beta (IL-1β), and human epididymis protein 4 (HE4), among others, in the context of CFTR modulation [61,62].

These markers reflect systemic and airway inflammation, as well as epithelial dysfunction, which are central features of CF pathophysiology. Initial findings suggest that CFTR modulator therapy can modulate these biomarkers in parallel with clinical improvements, providing additional insight into treatment response and potentially predicting long-term outcomes. As the field moves toward precision medicine, integrating such biomarkers into routine monitoring may offer a valuable complement to spirometry and imaging, enabling a more individualized approach to CF care.

Registry data consistently demonstrate reductions in healthcare resource utilization following CFTRm initiation, including decreased hospitalizations, outpatient visits, and the use of various symptomatic therapies. The findings of Kirwan et al. for IVA [26] and Bower et al. for ETI [59] document substantial reductions in hospitalizations and PEx, while the comprehensive analysis by Råket et al. provides detailed evidence of sustained reductions in multiple healthcare utilization metrics two years post-ETI initiation, including elective hospitalizations, outpatient contacts, and pharmacy visits [29].

The projected reduction in total population requirements for intravenous antibiotics with ETI, as estimated by Keogh et al. to be between 16.1% (~17,800 days) and 43.6% (~39,500 days), has significant implications for resource allocation and planning [28]. Similarly, the observed reductions in routine therapies, particularly airway medications and antibiotics, documented by Råket et al. two years after ETI initiation, reflect the profound impacts these modulators have on traditional treatment paradigms [30]. The divergence in treatment patterns noted by Granger et al. since the introduction of IVA provides evidence of evolving approaches to CF management in the modulator era, with potential reductions in symptomatic therapies as disease control improves [27]. These changes have important implications for long-term treatment burden, adherence, and quality of life.

The increased pregnancy rates among women taking CFTRms reported by Heltshe et al. reflect the profound impact that these therapies have had on life expectations and family planning decisions within the CF community. However, this trend also underscores the urgent need for more data on the safety of these medications during pregnancy and their potential effects on fetal development [31].

The quality of life improvements with ETI documented by Martin et al. in CF patients with advanced disease, including rapid and positive physical, psychological, and social effects translating into an improved quality of life and the formulation of new life goals, highlight the comprehensive impact of effective modulator therapy beyond physiological parameters [32]. These findings emphasize the importance of patient-reported outcomes in evaluating the full value of these therapies.

The comprehensive safety analysis by Ramsey et al., addressing concerns about depression symptoms and depression-related events in people with CF treated with ETI, provides reassurance that such events are generally consistent with the background epidemiology in the CF population and do not suggest a causal relationship with treatment [33]. This highlights the value of registry data for post-marketing safety surveillance and addressing emerging concerns. The observation by Burgel et al. that adults who discontinued LUM/IVA, often due to adverse events, were at a high risk of clinical deterioration emphasizes the importance of managing side effects and supporting treatment adherence to maintain clinical benefits [57]. This finding also highlights the need for alternative therapeutic options for patients who cannot tolerate specific modulators.

Registry studies have highlighted significant disparities in access to CFTRms based on geographic location, economic factors, and mutation patterns. The analyses by Guo et al., Tomlinson et al., and Kerem et al. emphasize the substantial unmet needs in many regions globally, particularly in low- and middle-income countries [45,46,47]. The findings of Cobanoglu et al. and Erdal et al. regarding modulator eligibility in Turkey (approximately one quarter and half of registered CF patients, respectively) highlight regional variations in mutation profiles compared to North American and European populations, underscoring the need for modulators effective against diverse mutations [43,49].

Even within countries where modulators are available, Hergenroeder et al. identified demographic and disease severity factors associated with delayed initiation, despite over 90% of eligible individuals being prescribed ETI within three years. This suggests inequities in access that require attention [42]. The projected negative impact of delayed access on lung health and survival, as modeled by Stanojevic et al., underscores the importance of addressing these disparities [39]. The characterization of the modulator-ineligible population by Sanders et al. highlights the continued need for therapeutic innovation to address patients who cannot benefit from current modulators due to rare or non-responsive mutations [44]. This population represents an important focus for future drug development efforts and clinical trials.

The projected impact of modulator therapies on survival is substantial, with López et al. suggesting that ETI treatment may allow people with CF to achieve a near-normal life expectancy, particularly with early initiation [40]. The validation of modeled survival projections for IVA against real-world registry data by McGarry et al. lends credibility to such long-term projections and supports their use in economic evaluations of these therapies [34]. Findings from regional studies, such as those by Kondratyeva et al. in Russia, provide valuable insights into population-specific responses to CFTRms and highlight the importance of local registry data in guiding the implementation and monitoring of these therapies in diverse healthcare contexts [37,60].

While CF registries provide invaluable real-world data, several limitations must be acknowledged. First, the observational nature of registry studies limits causal inferences compared to RCTs. Second, variations in data collection, definitions, and reporting practices across registries, as highlighted by Tomlinson et al., complicate cross-national comparisons and may introduce biases [46]. Third, registry data may underrepresent certain populations, particularly in regions with limited registry infrastructure or healthcare access. Inconsistencies between registries, such as those noted by Calthorpe et al. between US and UK data regarding PERT use following IVA, highlight the need for standardized approaches to data collection and reporting [20]. The poor consistency in registry reporting identified by Tomlinson et al. further emphasizes this need and calls for urgent action to create uniform reporting standards [46].

Although registries provide real-world data from large populations, dedicated cohort studies often offer a higher data quality, greater detail, and stronger evidence. Registry-based investigations offer several distinct advantages, including access to large sample sizes that enable the detection of modest treatment effects and the evaluation of rare CF genotypes. These studies capture real-world treatment effectiveness under routine clinical conditions, incorporating variations in adherence, concomitant therapies, and healthcare system differences. The longitudinal nature of CF registries facilitates assessments of long-term outcomes and temporal trends. However, registry studies face inherent limitations affecting their evidence quality. Data collection protocols vary between registries and may change over time, potentially introducing measurement bias. Substantial missing data rates, particularly for specialized outcomes, present analytical challenges. The observational nature precludes definitive causal inference, while non-randomized treatment assignment introduces potential selection and indication bias.

Dedicated cohort studies typically demonstrate a superior data quality and methodological rigor compared to registry analyses. These investigations employ standardized outcome measurements, comprehensive baseline characterization, and systematic follow-up protocols. The enhanced clinical detail enables sophisticated analyses of dose–response relationships, biomarker correlations, and adverse event profiles.

Well-designed cohort studies can implement robust analytical strategies, including matching, propensity score analyses, and instrumental variable approaches to minimize confounding. They provide opportunities for mechanistic investigations through specialized testing not routinely available in registry settings.

The evidence base demonstrates a remarkable consistency between registry-based and cohort study findings across key clinical outcomes, strengthening confidence in observed treatment effects. Regarding pulmonary function, both study types consistently demonstrate significant FEV_1_ improvements following CFTR modulator initiation. Registry studies document improvements ranging from 2–4% predicted for lumacaftor/ivacaftor to 10–14% predicted for elexacaftor/tezacaftor/ivacaftor in F508del patients. Cohort studies corroborate these findings while providing a more detailed characterization of response heterogeneity and modifying factors [63,64,65,66]. Registry analyses document 20–60% reductions in hospitalization rates and antibiotic treatment courses following modulator initiation. Cohort studies confirm these findings while providing additional insights into exacerbation severity, duration, and recovery patterns. Some registry studies with extended follow-ups suggest the potential waning of benefits over time, requiring confirmation through rigorous longitudinal cohort studies [67].

Despite substantial evidence from both approaches, important limitations persist. Long-term safety and effectiveness data remain incomplete, with most cohort studies having relatively short follow-up periods. Evidence for rare genotypes and special populations, including pregnant women and patients with advanced disease, remains limited. Neither study type has systematically incorporated emerging biomarkers for treatment response prediction or therapy optimization.

CFTR modulators have transformed CF care from reactive symptom management to proactive molecular-targeted therapy. This paradigm shift requires healthcare systems to develop competencies in genetic counseling, biomarker interpretation, and precision medicine implementation. Treatment algorithms now prioritize genotype-based therapy selection, with traditional therapies serving complementary roles.

The high acquisition costs necessitate careful evaluations of cost-effectiveness and healthcare resource allocation. Registry analyses suggest potential reductions in hospitalizations while increasing outpatient monitoring needs. Both study types identify concerning disparities in access and outcomes across different demographic and socioeconomic groups, requiring targeted interventions to ensure equitable access.

The convergence of evidence provides a foundation for future optimization through enhanced data integration strategies that leverage the complementary strengths of both approaches. Priority areas include precision medicine advancement through the integration of genetic, biomarker, and clinical data to develop predictive treatment response models. Systematic long-term follow-up studies combining registry infrastructure with enhanced data collection protocols could address critical knowledge gaps regarding durability, safety, and disease progression impact [68]. Future research using registry data should focus on several of the following key areas: long-term safety and effectiveness beyond 5–10 years, including potential effects on aging with CF; impacts on rare complications and extra pulmonary manifestations not captured in clinical trials; outcomes in populations typically excluded from trials, such as those with advanced disease or significant comorbidities; pregnancy outcomes and effects on offspring; the optimal timing of initiation and the potential benefits of early intervention; and the development of standardized international registry protocols to enable more robust global comparisons. Additional research priorities include investigating the mechanisms underlying nutritional changes with modulator therapy, understanding the differential effects on glucose metabolism in various stages of CFRD, exploring the potential for modulators to modify the disease course in patients with rare mutations, and evaluating the long-term impacts on antibiotic resistance patterns and microbiome composition.

## 5. Conclusions and Future Directions

Registry-based studies have confirmed and extended the clinical benefits of CFTRms observed in RCTs, demonstrating improvements across multiple domains, including pulmonary function, infection rates, nutritional status, healthcare utilization, and quality of life. These real-world data support the characterization of CFTRms, particularly the triple-combination therapy ETI, as disease-modifying treatments that have fundamentally altered the trajectory of CF. The profound implications for lung transplantation needs, survival projections, and healthcare resource utilization suggest that these therapies may substantially transform the epidemiology and management of CF in the coming decades. However, significant challenges remain in ensuring equitable global access to these transformative therapies and in addressing the needs of patients ineligible for current modulators. Continued investment in CF registries will be essential for monitoring long-term outcomes and guiding future therapeutic developments in this rapidly evolving field.

## Data Availability

Data are contained within the article.

## Abbreviations

The following abbreviations are used in this manuscript:

ABPA	Allergic
BA	Broncho-alveolar
BMI	Body mass index
CGM	Continuous glucose monitoring
CF	Cystic Fibrosis
CFRD	Cystic Fibrosis-related diabetes
CFTR	Cystic Fibrosis transmembrane conductance regulator
CFTRm	CFTR Modulator(s)
CT	Computed Tomography
ECFSPR	European Cystic Fibrosis Society Patient Registry
ETI	Elexacaftor/Tezacaftor/Ivacaftor
HbA1c	Hemoglobin A1c
IVA	Ivacaftor
LCI	Lung Clearance Index
LUM	Lumacaftor
MeSH	Medical Subject Headings
Pa	Pseudomonas aeruginosa
PERT	Pancreatic enzyme replacement therapy
PEx	Pulmonary Exacerbations
ppFEV_1_	percent predicted forced expiratory volume in one second
RCT	Randomized controlled trials
